# Correlation between α-SMA and ß-catenin levels in bronchoalveolar lavage fluid and severity of pneumonia

**DOI:** 10.12669/pjms.38.3.5329

**Published:** 2022

**Authors:** Xiaoying Li, Zinan Jiang

**Affiliations:** 1 Xiaoying Li, MD. Department of Emergency, The Third Xiangya Hospital, Central South University; No. 138, Tongzipo Road, Changsha 410013, Hunan Province, P.R. China; 2 Zinan Jiang, MD. Department of Emergency, The Third Xiangya Hospital, Central South University; No. 138, Tongzipo Road, Changsha 410013, Hunan Province, P.R. China

**Keywords:** Bronchoalveolar lavage fluid, α-smooth muscle actin, ß-catenin, Severe pneumonia, Retrospective analysis

## Abstract

**Objectives:**

To assess the association of bronchoalveolar lavage fluid (BALF) α-SMA and ß-catenin levels and the severity of pneumonia.

**Methods:**

The records of patients with severe pneumonia treated in our hospital from June 2019 to June 2020 were selected. The clinical outcome was observed within 10 days. For the purpose of analysis, patients were divided into two groups according to the outcome, 47 cases in the improvement group and 39 cases in the deterioration group. The intubation time, mechanical ventilation time and APACHE II score 10 days after admission were compared between the two groups; We assessed pulmonary infections using the clinical pulmonary infection score(CPIS). The levels of α-SMA and ß-catenin in bronchoalveolar lavage fluid at different time points were compared and analyzed, to analyze the association between the levels and the CPIS.

**Results:**

The APACHE II score in the improvement group were lower than those in the deterioration group (P<0.05). The expressions of α-SMA and ß-catenin in the BALF of patients in the improvement group were significantly lower than those of patients in the deterioration group on day 1, 3, and 7 (P<0.05); and the expressions of α-SMA and ß-catenin in the BALF of patients in the improvement group decreased with time, while those of patients in the deterioration group increased gradually with time(P<0.05). The expressions of α-SMA and ß-catenin in patients with CPIS>6 was significantly higher than those in patients with CPI≤6(P<0.05). Pearson correlation analysis showed that the levels of α-SMA and ß-catenin in BALF were positively correlated with the CPIS.

**Conclusion:**

The levels of α-SMA and ß-catenin in BALF are closely associated with the clinical condition of patients with severe pneumonia; the levels are positively associated with the severity of the disease and they increase with symptomatic worsening.

## INTRODUCTION

Severe pneumonia, an inflammatory pulmonary disease caused by bacteria or viruses, is common in ICU patients. Moreover, mechanical ventilation increases the risk of pulmonary infections. Medical advances and anti-infection treatments have improved gradually, but the incidence and mortality of severe pneumonia remain high.^1^,^2^ Therefore, assessing the clinical condition of patients with pneumonia is important to carry out prompt effective treatments that can improve their prognoses. Fiberoptic bronchoscope can suck and remove the secretion of airway under direct vision, and effectively remove the secretion of distal bronchus by lavage to relieve airway obstruction and restore ventilation. In addition, direct extraction of airway secretions for bacterial culture can reduce the chance of oral flora pollution, make the use of antibiotics more targeted, and improve the therapeutic effect of conventional drugs.^3^,^4^ Inflammation is important during the development of severe pneumonia and often leads to pulmonary fibrosis. Alpha smooth muscle actin (α-SMA) is a specific marker of myofibroblasts, and ß-catenin is a multifunctional protein that participates in the proliferation, differentiation, and migration of various types of cells. Both proteins are active during pulmonary fibrosis, but they are rarely used as clinical markers to assess the condition of patients with severe pneumonia. We hypothesized that the levels of α-SMA and ß-catenin in bronchoalveolar lavage fluid (BALF) may be associated with the severity of disease in patients with pneumonia. We designed a retrospective study using clinical records of 86 patients with severe pneumonia treated in our hospital. We assessed α-SMA and ß-catenin level changes in BALF samples and compared them to clinical data (including APACHE II and pulmonary infection scores) to explore the association between the expression of α-SMA and ß-catenin levels and the severity of pneumonia to provide guidance for patient management and prognoses.

Our objective was to assess the association of bronchoalveolar lavage fluid (BALF) α-SMA and ß-catenin levels and the severity of pneumonia.

## METHODS

We collected clinical data of 86 patients (46 men and 40 women) with severe pneumonia treated in our hospital from June 2019 to June 2020. We divided patients into improvement (47 cases) and deterioration (39 cases) groups based on their clinical outcomes.

### Inclusion Criteria:

- Patients older than 50 years with severe pneumonia diagnosed based on the criteria of the American Thoracic Society;
- Patients having received more than three treatments;
- Patients needing invasive ventilation or on septic shock;
- Patients having signed informed consent forms, giving permission to use their clinical data for research.

### Exclusion criteria:

- Patients with severe hypoxemia;
- Patients with coagulation dysfunction, immune dysfunction, immune deficiency, or immunosuppression;
- Patients with severe trauma, major operation, or long-term hormone therapy until the previous week;
- Patients with other severe infections or diseases.

The ethics committee of our hospital approved this study (No. 2021047, Date: 2021-07-22).

All patients underwent bronchoalveolar lavages on day 1, 3, and 7 after admission for a microbiological purpose, and to monitor risk of ventilator-associated pneumonia. The lavage site was the right middle or the left lingual lung lobe, and the operation was carried out following standard guidelines. Briefly, 150 ml sterile normal saline through the lavage catheter were used to lavage the bronchoalveoli through the fiberoptic bronchoscope three times, and a syringe was used for fluid recovery. After centrifugation, the BALF was stored at -80^0^ for later use. RT qPCR and Western blot were used to detect the expression of α-SMA and ß-catenin in alveolar lavage fluid.

We extracted the following clinical information from data of the two groups: a. Gender, age, tracheal intubation time, mechanical ventilation time, and chronic health score II (APACHE II) 10 days after admission ; b.α-SMA and ß-catenin levels from fiberoptic bronchoalveolar lavage results on day 1,3,and 7.We classified patients into two groups based on their clinical pulmonary infection score (CPIS) on the day of admission; those with CPIS>6 had a more severe pulmonary infection than those with CPIS ≤6.

### Statistical Analysis

We used SPSS 22.0 to analyze the data, the counting data are expressed as n (%) and were tested using *χ*^2^; the measurement data are expressed as ± SD and were tested using the t-test. We applied the Pearson correlation analysis for our correlation analysis considering P<0.05 as indicating a significant difference.

## RESULTS

A total of 86 patients met the inclusion criteria, including 48 males and 38 females; The age ranged from 55 to 79 years, with an average of (59.42±3.26) years. We found similar gender and age proportions in the two groups (P>0.05). The tracheal intubation and mechanical ventilation times, and the Apache II scores in the improvement group were lower than those in the deterioration group and the differences were statistically significant (P<0.05) Table-I

**Table I:** Comparison of basic data between the two groups(*x*¯±s).

Group	n(male/female)	Age(year)	Intubation time(hour)	Duration of mechanical ventilation (hour)	APECHE II score
Improvement group	47(25/22)	59.74±4.65	95.78±6.8	88.95±6.29	18.78±1.36
Deterioration group	39(21/18)	61.23±4.03	139.94±13.55	138.71±14.79	27.2±1.93
*t*	0.06	1.564	18.503	20.909	22.845
*P*	0.952	0.122	*P*<0.001	*P*<0.001	*P*<0.001

**Fig. 1:**
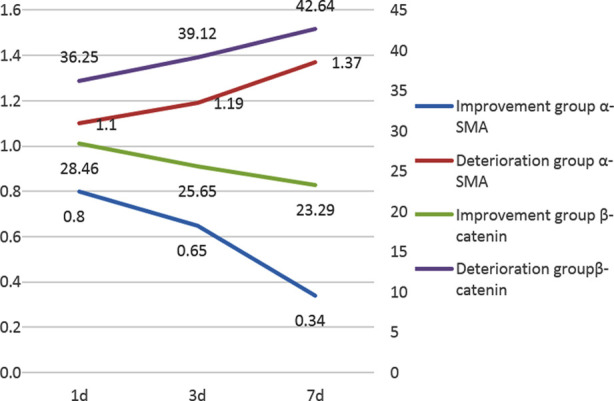
change curve of α- SMA and β- Catenin expression on day 1, day 3 and day 7 in two groups

The levels of α-SMA and ß-catenin in BALF of patients in the improvement group were significantly lower than those in the deterioration group on day 1, 3, and 7 (P<0.05); and the levels of α-SMA and ß-catenin in BALF of patients in the improvement group decreased over time, while the levels of patients in the deterioration group increased gradually over time(Fig. 1); the difference between the groups reached statistical significance (P<0.05) Table-II. The levels of α-SMA and ß-catenin in patients with CPIS >6 were significantly higher than those in patients with CPIS ≤6, and the difference was statistically significant (P<0.05) ~Table-III.

**Table II:** Comparison of the levels of α-SMA and ß-catenin in BALF in two groups at different time points (*x*¯±s).

Group	α-SMA(μg/L)			β-catenin(μg/L)		

	1st	3rd	7th	1st	3rd	7th
Improvement group	0.8±0.06	0.65±0.04^①^	0.34±0.05^①②^	28.46±1.62	25.65±1.11^①^	23.29±1.33^①②^
Deterioration group	1.1±0.08	1.19±0.08^①^	1.37±0.06^①②^	36.25±1.33	39.12±1.7^①^	42.64±1.2^①②^
*t*	19.453	37.317	84.084	23.962	42.459	69.976
*P*	*P*<0.001	*P*<0.001	*P*<0.001	*P*<0.001	*P*<0.001	*P*<0.001

Note: compared with the 1st day,

① P < 0.05; Compared with the 3rd day,

② P < 0.05.

**Table III:** Levels of α-SMA and ß-catenin in BALF of patients with different degrees of pulmonary infection (*x*¯±s).

Group	α-SMA(μg/L)	β-catenin(μg/L)
CPIS score<6(n=49)	0.9±0.046	32.51±0.98
CPIS score≥6(n=37)	1.02±0.02	34.27±0.8
t	15.985	8.882
P	P<0.001	P<0.001

Pearson correlation analysis showed that the levels of α-SMA and ß-catenin in BALF were positively correlated with the CPIS (r=0.842, P<0.001; and r=0.696, P<0.001, respectively).

## DISCUSSION

In this study, α-SMA and ß-catenin in bronchoalveolar lavage fluid were used as the evaluation indexes of patients with severe pneumonia. It was found that their levels were positively correlated with the severity of patients with severe pneumonia. Chen L^5^ et al. discussed the mechanism of thymocyte differentiation antigen-1(THY1) in acute interstitial pneumonia (AIP) affecting pulmonary fibrosis from the perspective of proliferation and apoptosis of mouse pulmonary fibroblasts. The results showed that pulmonary fibrosis was positively correlated with the phosphorylation of α-SMA and ß-catenin. It can be seen that the levels of α-SMA and ß-catenin can reflect the condition of patients with pneumonia. At the same time, studies have shown that in patients with severe pneumonia, inflammatory factors act on the alveolar membrane to cause damage, or inflammatory reactions mediated by inflammatory cells and their mediators lead to diffuse alveolar damage, which leads to the damage of alveolar epithelial cells and participates in the development of severe pneumonia.^6^,^7^

We divided patients into improvement and deterioration groups based on their clinical outcomes (the tracheal intubation time, mechanical ventilation times and the APACHE II score) . The levels of α-SMA and ß-catenin in BALF in the improvement group were significantly lower than those in the deterioration group on day 1, 3, and 7. In addition, the levels decreased with time in the improvement group and they increased in the deterioration Group (P<0.05). The increase in these markers indicates that the lung function of patients is seriously damaged and that they need endotracheal intubation and mechanical ventilation. However, endotracheal intubation and mechanical ventilation can worsen lung infection and reduce APACHE II score.^8^,^9^ Our finding of higher α-SMA and ß-catenin levels in the BALF of patients with deterioration suggest a correlation between the levels and the clinical condition. BALF contains a variety of soluble proteins derived from cells in the alveoli. This protein content changes with the number and function of cells in the alveoli and is altered during diseases causing lung lesions.^10^,^11^ α-SMA in BALF is derived from myofibroblasts that can replace alveolar epithelial cells, aggravating the destruction of alveoli and causing severe pneumonia. ß-catenin is an important epithelial cell adhesion factor expressed by fibroblasts, alveolar epithelial cells and smooth muscle cells during pathological processes. The ß-catenin signal promotes epithelial cell differentiation and injured cell healing, smooth muscle cells division, and proliferation of bronchial and alveolar epithelial cells. ß-catenin also inhibits the epithelial cells apoptosis and regulates the secretion of fibroblast-proliferation related factors, effectively regulating tissue fibrosis.^12^,^13^

By comparing the BALF levels of α-SMA and ß-catenin in patients with different degrees of pneumonia, we found that the levels in patients with CPIS >6 was significantly higher than that in patients with CPIS ≤6 (P<0.05). Pearson correlation analysis showed that the levels of α-SMA and ß-catenin in BALF were positively correlated with the CPIS (r=0.842, P<0.001 and r=0.696, P<0.001; respectively), indicating that the levels are closely related to the severity of pulmonary infection in these patients. CPIS is an important quantitative index of the degree of pulmonary infection. The score is directly proportional to the severity of the pulmonary infection, and a score>6 indicates the presence of a serious pulmonary infection. The levels of α-SMA and ß-catenin in patients with severe pneumonia with CPIS>6 were higher than those of patients with lower CPIS, suggesting that the levels of α-SMA and ß-catenin in BALF increased with the severity of the disease. This may be due to pulmonary fibroblasts proliferating, differentiating and migrating to promote the expression of α-SMA in the alveolar space, leading to the activation of myofibroblasts and the deposition of extracellular matrix in alveolar stroma. An excess of myofibroblasts will affect the lung repair function, affect the lung functions and aggravate the disease. Therefore, α-SMA levels in patients with severe pneumonia can reflect the activation of lung repair mechanisms. The higher the α-SMA level, the worse the damage to the lung tissue, an important consideration for prognosis evaluation.^14^,^15^ ß-catenin is a multifunctional protein expressed mainly in stroma and epithelial cells. Under normal conditions, ß-catenin is degraded by proteases. However, WnT signal activation during damage to the epithelium results in accumulation of phosphorylated ß-catenin in the cytoplasm. The accumulated ß-catenin protein will penetrate into the nucleus, regulate the expression of target proteins, and promote the activation of pulmonary fibroblasts. This promotes pulmonary fibrosis and pneumonia aggravation.^16^,^17^ Blockage of the ß-catenin signal transduction pathway may inhibit fibrosis and this treatment idea may help improve the outcome of patients with severe pneumonia.

### Limitations of the study

First, the study was not extensive and relied on retrospective analysis. Second, the sample size is small. Nevertheless, it is necessary to further accumulate relevant data and conduct more in-depth and detailed research on the condition of patients with severe pneumonia in the future.

## CONCLUSION

The levels of α-SMA and ß-catenin in BALF were positively correlated with the severity of pneumonia and may become prognosis and treatment management markers for patients with severe pneumonia.

### Authors’ contributions:

**XL:** conceived and designed the study.

**XL and ZJ** collected the data and performed the analysis.

**XL:** was involved in the Writing of the manuscript and is responsible for integrity of the study.

All authors have read and approved the final manuscript.
